# Critical Thinking Emulation Model With Outcomes‐Based Assessment

**DOI:** 10.1002/jdd.70171

**Published:** 2026-02-12

**Authors:** David C Johnsen, Leonardo Marchini, Carlos Garaicoa‐Pazmino, Aditi Jain, John Syrbu, Matthew Geneser, Azeez Butali, Jennifer E Hartshorn, Jhanvi Desai, Clark M Stanford, Kim Vo, Michael Spector, Nicole McGlynn, L Brendan Young

**Affiliations:** ^1^ Pediatric Dentistry College of Dentistry University of Iowa Iowa City Iowa USA; ^2^ Preventive and Community Dentistry College of Dentistry University of Iowa Iowa City Iowa USA; ^3^ Periodontics College of Dentistry University of Iowa Iowa City Iowa USA; ^4^ Research Center School of Dentistry Universidad de Especialidades Espiritu Santo Samborondón Ecuador; ^5^ Operative Dentistry College of Dentistry University of Iowa Iowa City Iowa USA; ^6^ Family Dentistry College of Dentistry University of Iowa Iowa City Iowa USA; ^7^ Oral Pathology, Radiology, and Medicine Iowa Oral Health Research Institute College of Dentistry University of Iowa Iowa City Iowa USA; ^8^ Prosthodontics College of Dentistry University of Iowa Iowa City Iowa USA; ^9^ College of Dentistry University of Iowa Iowa City Iowa USA; ^10^ College of Dentistry University of Iowa Iowa City Iowa USA

**Keywords:** critical thinking, outcomes‐based assessment, symbiotic learning network

## Abstract

**Background/Purpose:**

Gaps exist between critical thinking importance and an organized agenda for the development of critical thinking learning outcomes. Multiple critical thinking skill sets were developed by the authors, forming a symbiotic network. The key concept for each critical thinking exercise follows one learning model emulating the master clinician's thinking. The purpose is to enhance symbiosis in a pedagogical framework for individual exercises with a common outcomes‐based assessment; internalization of each skill set is followed by nuance exploration—“learning moments.”

**Methods:**

Descriptive analyses for (1) a symbiotic network of patient‐based, student‐led demonstrations of thinking and judgment with expanded opportunities for critical thinking, (2) a common outcomes‐based assessment for patient‐based student‐led demonstrations of thinking and judgment, and (3) learning moments for each skill set: periodontics, operative dentistry (caries), geriatrics, ethics, pediatrics, TMD, and technology decision‐making.

**Results:**

Two factors were seen to contribute to interactions/sharing/symbiosis of learning guides among different departments: using a common emulation‐learning model and using a common outcomes‐based assessment. The use of a learning guide based on a designated thought process, combined with the use of a common outcomes‐based assessment were also associated with subsequent rich and varied learning moments distinctive for each exercise.

**Conclusions:**

With little literature on learning outcomes for critical thinking, the development of an emulation learning model based on the thought processes of the master clinician led to the development initially of multiple critical thinking skill sets, followed by interactions of exercises. The result was a symbiotic network with rigorous demonstration of a skill set followed by a rich exploration of nuances.

## 1 Introduction

Despite broad agreement on the importance of critical thinking in dental education, a clear and systematic agenda for developing critical thinking learning outcomes remains underdeveloped [1, 2, 3, 4]. Meaningful progress requires active discussion about how learning outcomes, learning guides, and assessment strategies for critical thinking should be structured. However, published guidance on articulating such outcomes and instructional methods is sparse and largely limited to reports from the present authors [5, 6, 7, 8].

There is little evidence describing how learning outcomes should progress from introductory concepts to guided learning, outcomes‐based assessment, and, ultimately, the critical thinking expected in clinical practice. An initial step is to establish the institutional importance for critical thinking as part of being a dentist. When the institutional priority is high, establishing learning outcomes for critical thinking would logically follow. In practice, this sequence rarely occurs.

A key strategy for reducing the gap between the perceived importance of critical thinking and the absence of structured learning outcomes is to emulate the intended activity of the student, specifically, the thinking the student is expected to demonstrate in patient care. The most direct way to accomplish this is to articulate the expert clinician's thought process for each critical thinking activity [5, 6, 7, 8]. For educators, the task is to distill the expert clinician's thought process into a concise, teachable sequence that novice clinicians, like students, can apply to their next patient encounter. This derivation of expert thinking is the most intellectually challenging step in developing critical thinking skill sets.

The approach parallels how psychomotor skills are taught. For a crown preparation, the faculty demonstrate what is expected, and students then perform the procedure. For clinical reasoning, students are similarly given the thought process of the expert and then asked to demonstrate that thought process with their patient. For each critical thinking skill set, the expert's thought process becomes the learning guide, and the basis for assessment of the learning outcome [5, 6]. We refer to this explicit approach as an *emulation‐learning model*, in which the derived expert thought process is sufficiently succinct for students to apply independently during patient care.

A network of patient‐based, student‐led demonstrations of clinical thinking and judgment—each guided by the same emulation‐learning model—creates a symbiotic structure for critical thinking instruction that can be applied across clinical departments [5, 7, 8, 9, 10]. When assessment functions as a continuation of the learning outcome rather than a separate “pass/fail” activity, the instructional sequence begins and ends with the same defined thought process.

Using a single assessment model for multiple critical thinking skill sets offers coherence across exercises and open opportunities eliciting the student to extend the act of critical thinking into nuances beyond the basic skill set. We refer to these “nuances” that are reflective, patient‐specific insights as *learning moments*.

The primary purpose of this project is to examine the feasibility of consolidating several existing critical thinking exercises into a unified, symbiotic network guided by a single emulation‐learning model. Such consolidation could enable a common outcomes‐based assessment that extends directly from the learning outcome itself. A secondary purpose is to explore whether completing the basic thought process steps opens opportunities for students and faculty to identify nuanced considerations, “learning moments,” unique to each patient. We hypothesized that (1) a shared structured outcomes‐based assessment would enhance integration across the critical thinking exercises, and (2) internalization of the thought process steps would enable deeper exploration of patient‐specific nuances.

Throughout the manuscript, the phrase **“**critical thinking skill set**”** is used as a *patient‐based, student‐led demonstration of thinking and judgment*. The term “exercise” is used interchangeably. Although aspects of this work have been previously published [9, 10, 11, 12, 13, 14, 15, 16], these prior studies laid the groundwork for consistency in outcomes‐based assessment and for learning moments.

## 2 Methods

This project was designated exempt by the University of Iowa Institutional Review Board (IRB #202501127) as it does not meet the regulatory definition of human subjects research and does not require review by the IRB. There was no experimentation with students; assignment of students to respective seminars was done in advance of the project. Faculty facilitated seminars in the same setting as before the project began.

This work is presented as a conceptual framework and feasibility study, and not a general model, examining whether multiple critical thinking skill sets can be consolidated under a single emulation learning model and documenting learning moments. Data from the project are available.

Eighty dental students were enrolled in the course. For each critical thinking skill set, materials were used from approximately half the class (*n* = 40) for analysis. Students were assigned to seminars before the study began. Seminars included approximately 10 students and were facilitated by trained faculty that were familiar with the emulation model. Students received a PowerPoint template aligned with the learning guide for each critical thinking skill set. After performing a comprehensive medical and dental evaluation, students entered required information into the template and delivered a 15‐min patient presentation. The instructional and assessment environment for critical thinking skill sets has been described previously shown [7, 8, 9, 10].

Table 1 provides an example of the combined learning guide for caries and periodontal risk assessment.

**TABLE 1:** Example of content for a learning guide and assessment instrument combined for periodontitis and caries risk analysis.

**Patient history**
Chief concern
Medications
Medical Hx (diabetes/smoking/obesity)
Dental Hx
Social Hx
Transportation/finances/support
Domestic stability/instability
**Patient examination**
Clinical exam
Radiographic exam
Interdisciplinary
**Which data are more important? Why?**
**Risk assessment**
Compliant/erratic/non‐compliant
Social barriers
Visible disease: present/not visible
Risk/progression
Minimum risk/minimum disease
High risk/minimum disease
High risk/progressing disease
ROHD
Diet/nutrition/behavioral: controlled/uncontrolled
**Diagnosis: prioritize conditions**
**Etiologic factors**
**What if we do nothing: 6 months/5 years**
**Treatment**
Rationale for selected Tx
Duration
Prognosis (anticipated Tx in 5 years)
Favorable/favorable with compliance/questionable/hopeless
Alternatives
**Evaluation of treatment outcomes**
Expected vs final outcome
Appropriate/successful
**Evidence‐based dentistry**
Ask: PICO question: comparative
Acquire
Appraise
Apply
Assess
**Summary**:
Alignment: compliance/risk/disease progression/prognosis
Will treatment likely bring health?
**Self‐assessment/biases**

To protect patient confidentiality, a faculty member covered identifying data with opaque tape. Deidentified copies were produced to ensure no identifiers could be recovered.

Two descriptive perspectives were examined:

**Integration of critical thinking skill sets into a symbiotic learning network**

Analyses focused on:

• Commonalities for current critical thinking skill sets
• Accommodation of new critical thinking skill sets using an emulation learning model
• High levels of performance by students
• Dynamics of exploring alternatives versus a “right” answer
• A succinct outcomes‐based assessment with a rationale for consolidating the main concepts of learning guidance and performance assessment
• Overall assessment of competency in critical thinking for dental practice

**Learning moments captured by faculty**

Learning moments were collected for the following critical thinking skill sets:

• Periodontal risk
• Caries risk
• Geriatrics
• Ethics
• Pediatrics
• Temporomandibular disorders (TMD)
• Technology decision‐making

Learning moments occurred as spontaneous insights or questions after a student completed the designated thought process steps measured by the outcomes‐based assessment for student performance rubric (Table 2). These remarks reflect expert reasoning about nuanced, patient‐specific considerations. After each student presentation, faculty explore nuances outside the learning guide and specific to the respective patient. While each faculty statement is spontaneous, some faculty comments almost always happen. Knowing that a faculty comment is coming, the recording faculty is ready to try to capture the main points of the faculty colleague.

**TABLE 2:** Outcomes‐based assessment rubric for student performance rubric.

Overall progress (concurrent with stage of education) to mastery/internalization for this skill set	Yes	No
Proactive assessment for each exercise		
Applied the steps for the designated thought process		
Explored viable alternatives		
Reactive assessment to deviations from ideal		
No remediation		
Remediation on the spot		
Separate remediation		
Learning moment		
Expand from basic analysis to nuance		

To record them:

- One faculty member hand‐recorded the key message(s) from attending faculty.
- Recordings captured the essence of the insight rather than verbatim phrasing.
- Faculty compared their handwritten entries of learning moments with other faculty to confirm agreement on the intended meaning.
- This strategy was used consistently across seminars, although the speed of recording limited completeness. Because this feasibility study focused on the process rather than formal qualitative analysis, no full coding framework or thematic saturation procedures were used. Instead, learning moments are summarized into descriptive patterns (Table 4).

Two examples (generalized to protect identity) include:

- “Periodontitis and A1C level: High risk with minimal current disease. Why?”
- “Important data? Details on diet—sweets; patient on cusp of change; treating the teeth or the person?”

## 3 Results

Results are descriptive from two perspectives: one perspective analyzed the dynamics of the symbiotic network of critical thinking skill sets. The second perspective analyzed “learning moments” for nuance exploration for each exercise.

*Dynamics of the symbiotic network of critical thinking skill sets*: concepts for critical thinking are the basis for each of the following six sections and appear as part of the transition from theory to practice: commonalities for current critical thinking skill sets; accommodation of new critical thinking skill sets using an emulation learning model; high levels of performance by students and faculty calibration; dynamics of exploring alternatives versus a “right” answer; A succinct outcomes‐based assessment with rationale for consolidating main concepts of learning guidance and performance assessment; and overall assessment of competency in critical thinking for dental practice.

### 3.1 Commonalities for Current Critical Thinking Skill Sets

A concept in the education literature is to engage the student in multiple experiences reflecting critical thinking, with the more such experiences, the easier it is to adapt to the next one. The network of critical thinking skill sets followed separate development of individual skill sets; each exercise with 10 students was repeated, usually four to eight times, with different students to accommodate 40–80 students. The design is consistent with the concept of multiple experiences (periodontics, caries/operative, geriatrics, technology decisions, TMD, pediatrics, ethics), making the next experience easier to adapt to; the extension of that concept is that the student will adapt to the next experience in practice. Only after several skill sets were developed were the benefits for a symbiotic association observed.

The phrase “symbiotic learning network” refers to a pedagogical framework; the phrase means that individual demonstrations of critical thinking exercises use the same learning model to reinforce the way of learning, as well as reinforce the effect of different and overlapping skill sets. Some of the learning guides overlapped, for example, the question, “Which of the data are most important and why?” came from the interprofessional practice (IPP) question and appears in five different skill sets; a consistent evidence‐based dentistry (EBD) guide was used in four skill sets. Two levels of internalization occur as the student demonstrates the thinking and judgment of the designated thought process: one level is “application” of steps in the designated thought process. Did the student apply the steps in the designated thought process? The “application” of steps is more objective; a deeper level is “grasped” the concepts in respective steps in the thought process. The “grasped” component is more subjective, although over time the level of agreement of faculty increases, but not to the levels of “applied” the steps. The ideas of assessment using “A” for applied the step and “G” for grasped the step are used with minor variation in each of the skill sets. The number of “A's” and “G's” in each skill set makes a single determination for student internalization incomplete without a final “Yes” or “No”.

### 3.2 Accommodation of New Critical Thinking Skill Sets Using an Emulation Learning Model

While many critical thinking skill sets have been developed using an emulation model, the question arises on the accommodation of additional critical thinking skill sets and whether a different learning model is needed. Thus far, each new critical thinking skill set has been accommodated using an emulation‐learning model shown in Figure 1. When this project started, we had no patient‐based, student‐led learning exercise for TMD. An exercise has been developed using standardized patients; the basic skill set is offered as an example; other skill sets have been reported previously. Basic data are given to the student. For the patient/case, the student is given health, dental, social history, oral exam, and photos to guide responses to questions in Table 3.

**FIGURE 1:**
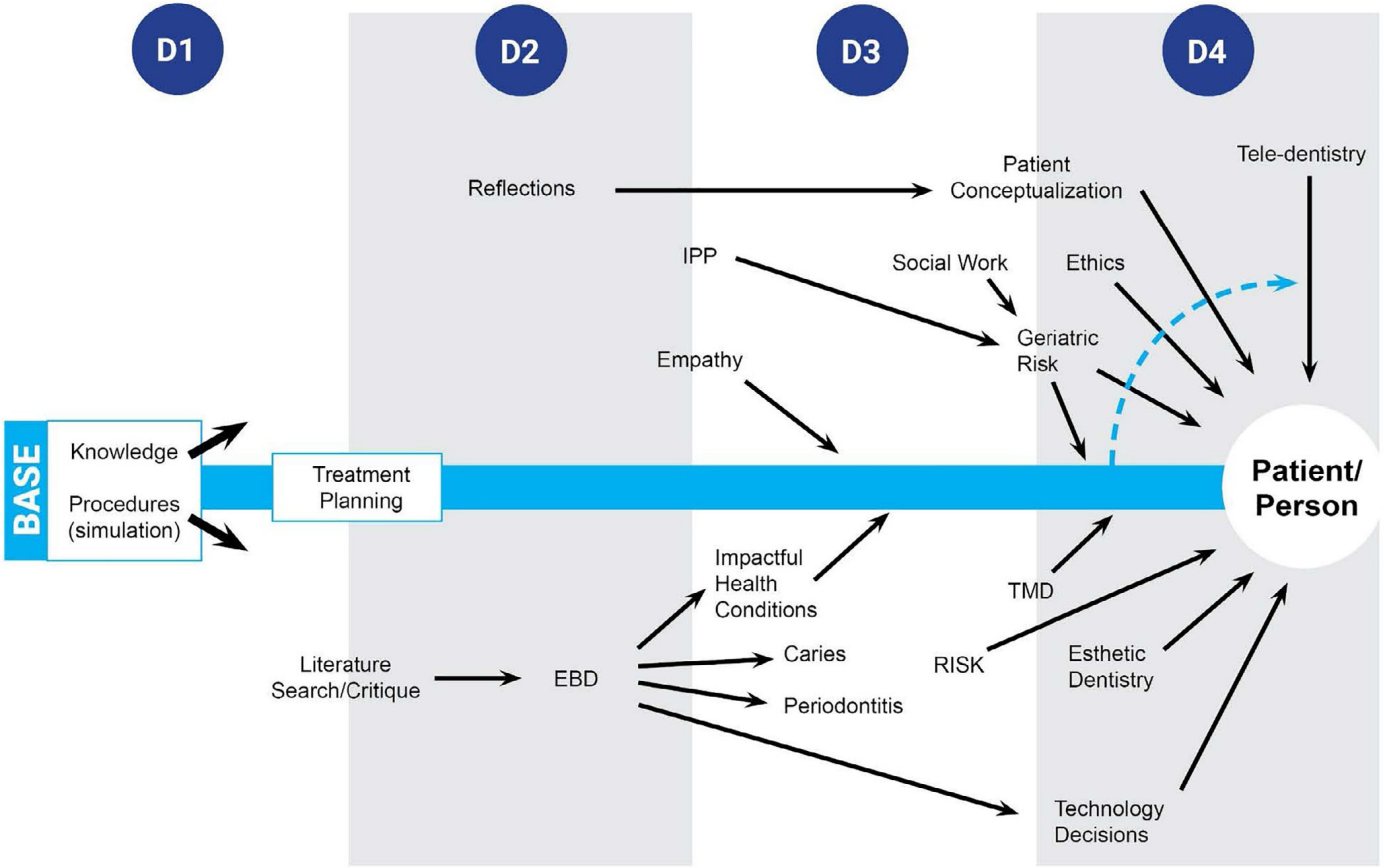
Schematic of a symbiotic network of patient‐based, student‐led demonstrations of thinking and judgment. Each critical thinking exercise is based on the thought process of the master clinician as the learning outcome, learning guide, and assessment instrument.

**TABLE 3:** Learning guide/assessment for temporomandibular disorder (TMD).

Learning guide/assessment for temporomandibular disorders (TMD)
Do we have the data for decisions?
What data are important and why?
What is the source of orofacial pain?
1. *Odontogenic pain*
2. *Predominantly muscular pain, with little or no temporomandibular joint (TMJ) involvement*
3. *Predominantly TMJ pain, with reduced muscular involvement*
4. *Pain from different origin: neurologic, otologic, trigeminal neuralgia, other/idiopathic*
5. *No pain with signs of TMJ involvement*
What is your rationale for this diagnosis?
What will happen if I do nothing?
What are treatment alternatives?
What are possible outcomes with and without different types of intervention?
What is the patient capacity for self‐management?
For each intervention, identify/offer evidence and rationale (including referral or no treatment)
Recommend specific interventions
Develop a communication plan
Self‐assess

All steps except for the third bullet, “What is the source of orofacial pain?” are used in other skill sets. The TMD exercise is the only exercise with an algorithm in the learning template, leading to a “Diagnostic Criteria for TMD” and a “diagnostic decision tree.” It may be worth noting that the TMD exercise differs from other critical thinking exercises in that TMD is the only one that is more of a developmental disorder and not (for example) a microbial disease, as are caries and periodontitis. Assessment is identical to the other patient‐based, student‐led demonstrations of thinking and judgment.

### 3.3 High Levels of Performance by Students

High levels of student performance have been reported in patient‐based student‐led demonstrations of thinking and judgment.

To explore potential factors: the use of alternatives in patient situations prompts the student to develop an array of possibilities for their patient; as long as the student explores alternatives, the act of critical thinking, emulating practice, is happening, and the student is encouraged to continue. The opportunity for remediation on the spot is an unexpected benefit in working with critical thinking skill sets since, at the point of presentation, there are rarely irreversible moments. Faculty can point out divergent directions in patient analysis thus, making this line of inquiry different from technical procedures, which can be irreversible. It is unusual to rare for a student following the learning template to have a gross deviation from patient standards. No remediation or remediation on the spot is more common. Much of critical thinking remediation happens on the spot.

### 3.4 Dynamics of Exploring Alternatives Versus a “Right” Answer

Two factors offer a level of assessment precision to determine adequate performance but limit the level of assessment precision to rank a large number of students; the two factors are the basic definition of critical thinking as an “art” and the thought process leading to alternatives [12]. The “art” of analyzing one's thought process with the intent of improving it leads to the discussion of how precisely “art” can be quantified. The exploration of alternatives is logical for patient care since there are rarely “right” answers.

### 3.5 A Succinct Outcomes‐Based Assessment With Rationale for Consolidating Main Concepts of Learning Guidance and Performance Assessment

Assessing performance follows the progression from first establishing the learning outcome, followed by guiding learning based on the outcome, and ultimately, assessing performance based on key components of the learning outcome.

Use of a single assessment as an extension of the learning outcome brings further symbiosis to the learning exercises and simplifies the process for faculty and students. Reinforcing two main components of critical thinking exercises—applying the steps in the designated thought process and exploring alternatives—brings a succinct conclusion to the learning outcome while providing an administrative outcome that the student successfully completed the exercise.

### 3.6 Overall Assessment of Competency in Critical Thinking for Dental Practice

One model of learning followed by one model of assessment can offer the opportunity for overall competency in critical thinking based on internalization of over a dozen critical thinking skill sets. While the domains are different and the (often overlapping) line of thinking is similar, the content is different. Many questions are the same from one exercise to another. As pointed out in the example of TMD, several questions are the same as for caries risk, periodontitis, risk, and geriatric risk.

A potential benefit for following this line of inquiry is that multiple critical thinking exercises can use an emulation model to guide learning with a single assessment model; thus, if an assessment model for critical thinking for patient‐based, student‐led exercises is identified, that model could serve as the assessment for a large number of critical thinking learning exercises. Critical thinking can then be assessed with one format across departments and dental school years.

*The second perspective analyzed “learning moments” for each exercise*: periodontitis risk, caries risk, geriatrics risk, ethics, pediatrics, technology decision‐making, and temporomandibular disorder (Figure 2).

**FIGURE 2:**
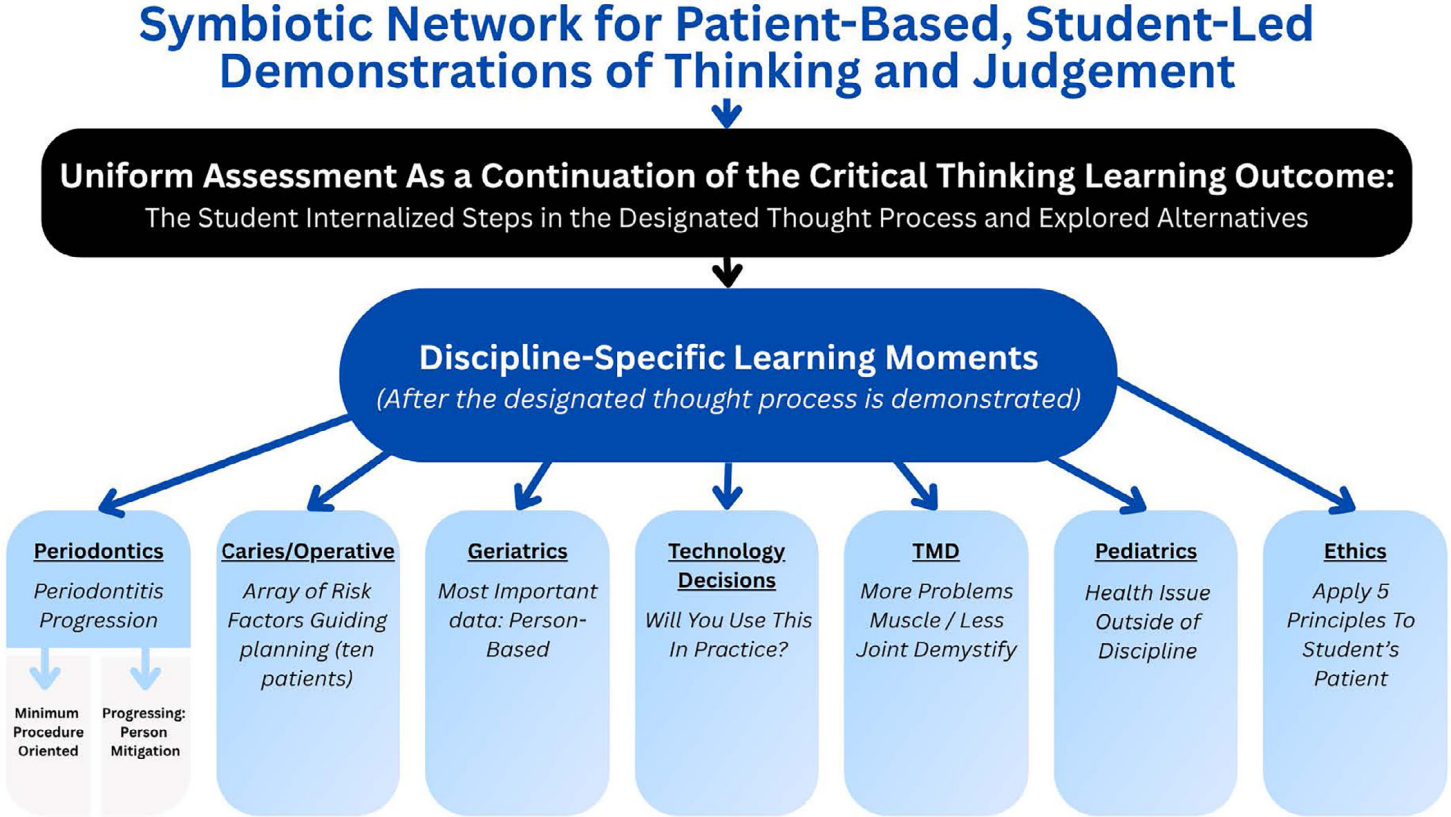
With each exercise using the same emulation‐learning model and by sharing ideas on learning guidance, individual exercises became a symbiotic network of over a dozen exercises. Similarities in assessment evolved with a commonality of successful completion of the exercise or not. The next evolutionary step underway is to assess performance as a continuation of the critical thinking learning outcome. Once the basic skill set is demonstrated (and only then), the dynamic is open for learning moments initiated by faculty to explore nuances for the patient or case. Patterns of learning moments are distinct for each skill set. Patterns for learning moments are elaborated in the text.

The phrase “learning moment” refers to reflective and reactive insights of faculty and students regarding nuances for the specific patient following (and not until) demonstration of the designated thought process. Learning moments are a natural momentum of the exercise, are not part of the assessment, and occur in nearly all the student presentations. Nuance exploration is an activity of expert. Nuance Exploration is a concept without much exploration. In this project, we were experimenting with ways to record nuance exploration by faculty. A first step was to have one faculty write the key points from a faculty comment following the student presentation. Notes on learning moments recorded by one faculty were collated for each of the seven exercises. For each exercise, a meeting was then held to include the faculty involved in the respective exercise. A preliminary overview of recordings was sequentially coalesced to determine patterns of responses. Patterns of responses were clear for each exercise. Patterns for learning moments were determined after recordings were collated. The next step was to gain the agreement of the faculty on a label for each response pattern. Response patterns are shown in Table 4 and Figure 2. With the distinct promise shown from nuance exploration (learning moments), the next step is to find ways to record the main ideas of faculty comments more completely and to look for ways to quantify response patterns. Assessment is based on the demonstration of the designated thought process and not on the learning moments. Some of the richest discussion occurs in learning moments after the students have demonstrated internalization of the designated thought process. The exploration of nuances does not happen until and unless the student demonstrates the designated thought process/analysis for their patient. The mechanism for implementation of the “learning moment” follows faculty commentary and questions for the student's presentation. Faculty and student comments are recorded by hand. “Learning moments” are collected for each seminar with more recordings in seminars where two faculty are present. Prominent patterns for comments emerged quickly for respective seminars. For each exercise, the sample size is 40. Commentary on examples for learning moments are summarized in Table 4. The rich range of topics was extensive and can be the subject of a separate project once this format was formalized. The “learning moments” approach shows great promise limited only be the speed the faculty member can record initial comments following each presentation.

**TABLE 4:** Sample of “learning moments.”

Learning moments	**Primary focus**	**Patterns**	**Takeaway**
Periodontics	Minimum disease vs. progressing disease	Minimum disease → focus on procedures, progressing disease → focus on mitigating periodontitis	Adjust teaching approach to tie interventions more closely with disease progression
Operative (caries)	Minimum disease vs. progressing disease	Stable care → reinforce minor interventions and compliance, extensive caries → emergency focus	Opportunity to discuss social factors (diet, home life, compliance) impacting disease
Geriatrics and special needs	Understanding and prioritizing multifactorial data that affects a patient	Focus less on teeth, more on mobility, POA, transport, compliance	Prioritize data emphasized social/health factors with less on dentition
Ethics	Applied ADA ethic principles to personal situations in each patient	Ethics discussion tailored to patient context (billing, abuse, bad news)	Alums report applying this directly in practice; emphasized real‐world applicability
Pediatric	Analyzing “impactful conditions” affecting health	Mix of caries and systemic and developmental conditions	Interdisciplinary thinking
Temporomandibular disorders	Conservative, patient‐led management	Empower patient through predominantly muscle pain	Newest exercise still expanding
Technology decision making	Decision‐making framework for adopting dental technologies	Responses vary widely	Student exercise encouraged deeper analysis, and alums reinforce further analysis beyond college education

## 4 Discussion

Internalization for multiple critical thinking skill sets has impacted a student learning process as demonstrated from (1) implementation of an array of patient‐based, student‐led demonstrations of thinking and judgment [6, 7, 8, 9, 10, 11]. Each exercise uses an emulation‐learning model to enhance a symbiotic network of critical thinking skill sets, (2) use of a single outcomes‐based assessment for each critical thinking exercise, and (3) the benefit from formalizing the “learning moment” to systematically explore nuances once the designated thought process is demonstrated. Each of these discussion points seems worthy of expanded exploration. The benefits of this continuity seem strong for the student who now has one familiar emulation‐learning model to use. The symbiotic dynamic with the network of critical thinking exercises provides a link for different patient analyses. The focus on learning dynamics is different from solving logistical problems such as scheduling, room assignments, accreditation assignments, etc. In addition to a uniform format, seeing questions that appear in multiple disciplines offers the student the chance to concentrate on the thought process more than logistics. An example is the consolidation of the learning guide for risk assessment for caries and periodontitis [10]. Other examples are social work and IPP incorporated into geriatrics; and EBD incorporated onto four skill sets [13, 14]. In addition, the knowledge that the thought processes will be used in practice raises interest for the student and reinforces concepts for use in practice [5]. The emulation model is scalable to any institution because all dental schools have outstanding teachers with valuable intuitive thought processes that have not been derived succinctly for use by students. Each skill set takes a couple of years to develop and mature; a symbiotic network takes several years.

Use of a single assessment as an extension of the learning outcome brings further symbiosis to the learning and simplifies the process for faculty and students. Reinforcing two main components of critical thinking exercises into assessments—applying the steps in the designated thought process and exploring alternatives—brings a succinct conclusion to the learning outcome. The assessment model also provides an administrative outcome that the student successfully completed the exercise.

Formalizing the “learning moment” systematically offers insights beyond the standard didactics. Feedback from a collection of learning moments can also offer the chance to adjust didactic instruction. Distinct patterns of learning moments for each exercise are not surprising. The patterns of nuance explorations are strikingly different from one exercise to another and can warrant further study. For example, the distinct differences between nuance explorations for periodontics patients with progressing disease and minimum disease are interpreted to indicate an intuitive judgment by faculty to focus on disease mitigation for patients with progressing disease and assume that the disease is not progressing to focus on procedures to bring health—implants, crown lengthening, etc. What is enlightening is the analysis and comparison of the learning moment patterns for respective exercises. Each nuance would likely not happen without a formal notation of faculty comments. The stage is then set for expansive exploration of nuances, when and only when the student demonstrates internalization of the basic designated thought process applied to their patient. We see the process of giving structure to something as unstructured as nuance exploration as a challenge to be advanced in a future project. We do see potential for giving structure to nuance exploration. After recording nuance explorations in a variety of seminars, the array of patterns distinct to each seminar may be worth a separate project. An opportunity arises in the setting with multiple presentations (many exercises have 10 students) to compare learning moments, for example, in patients with a disease, “How would you compare the risk levels of the first four patients we analyzed today?” We submit that the carryover to practice is reinforced with multiple experiences reflecting critical thinking.

Several factors may explain the high levels of performance: the use of alternatives more than “right” answers, the opportunity for remediation on the spot; the belief that these skills will be used in practice to make them a better dentist; the use of a template with open ended questions; continuity of questions from one skill set to another; and presenting in front of faculty and peers [5].

While assessing each step in a designated thought process offers completeness, the number of steps (some with 20 or more) in each thought process can make assessment cumbersome; thus, the focus of a summary assessment in concert with the learning outcome. High agreement of faculty on assessing performance has explanations. First, faculty have been using the basic learning guide/assessment instrument for several years and have spot checked levels of agreement each year. Second, it is the regular editing of the learning guide for completeness, combined with succinctness—what are the fewest words to gain the main points? Thus, faculty have a guide that is both familiar and easy on the eyes.

Surveys of alumni have shown that students use patient‐based, student‐led demonstrations of thinking and judgment in practice, and students regularly give high scores in post‐course feedback on the potential value for practice; thus, students take the experience seriously with the belief that the skill will make them a better dentist.

Assessing student choices of alternatives has limits in precision. Encouragement to explore alternatives leads the student to analyze each alternative—a basic critical thinking exercise in patient care. Funneling the student to a “right” answer, as in a diagnostic decision tree, is different from exploring alternatives with a patient. Thus, the limits on precision on student exploration of alternatives.

The challenge is raised whether competency for critical thinking for patient‐based, student‐led exercises can be meaningfully assessed in a single high‐stakes assessment. Thus, the same assessment is used over a network of a dozen exercises. Repetition of the same assessment sets the stage or overall declaration of mastery (internalization) of critical thinking in patient related situations. Thus, overall competence in critical thinking relative to patient care could be based on over a dozen student demonstrations in the act of critical thinking rather than a single high‐stakes exam, the validity of which (the latter) could be in question.

### 4.1 Limitations

With little literature on learning outcomes for critical thinking, comparisons are limited. Since critical thinking is an “art” and while performance can be assessed at/to a level of internalization (interpreted as mastery) for an exercise, comparisons are challenging with knowledge and technical (procedural knowledge), where greater precision is possible [15]. A limitation is that much of the literature on critical thinking, specifically patient‐based, student‐led demonstrations of thinking and judgment, has been gathered by the current group. Thus, comparisons are limited, leaving comparisons with previous literature from the current group. Accepting that different domains will have different learning outcomes, learning guidance, and assessment is an inherent part of overall competency assessment for practice and the topic of a different story [15]. The hope is that the paper will elicit further exploration of learning outcomes, learning guidance, and outcomes‐based assessment for critical thinking.

### 4.2 Summary

(1) The dynamic of a symbiotic network for patient‐based, student‐led demonstrations for thinking and judgment presents opportunities for expanded student learning by consolidating ongoing exercises with a single emulation‐learning model. A common learning model based on emulation allows several points of consolidation without disrupting the autonomy of the respective skill set. (2) A common performance assessment is feasible for each of the patient‐based, student‐led demonstrations of thinking and judgment. The assessment completes the progression of the learning outcome and is not a separate activity. (3) The single assessment sets the stage for faculty and students to pursue nuances beyond standard didactics once/after the student has demonstrated the basics of the learning exercise. Exploring nuances was one of the most exciting parts of the project. The “learning moments” approach shows great promise limited only be the speed the faculty member can record initial comments following each presentation.

## Author Contributions

David C Johnsen initiated the plan for the project, engaged students in each of the exercises multiple times, and, for most exercises, for multiple years, designed the plan for the initial manuscript and drafted the initial manuscript. Leonardo Marchini designed three of the exercises, engaged students in three of the exercises, and aided in the design of the project and the initial manuscript. Carlos Garaicoa, Aditi Jain, John Syrbu, Matthew Geneser, Azeez Butali, Jennifer Hartshorn, and Jhanvi Desai designed and administered respective exercises, delivered didactic material in preparation for exercises, engaged students in respective exercises, gave periodic input as the project progressed, and reviewed drafts of the manuscript. Kim Vo assembled data for the project, reviewed de‐identified data for each of the exercises, and assisted in the design of the initial manuscript draft. Clark M Stanford delivered didactic material for one of the exercises, engaged students in one of the respective seminars, and reviewed drafts of the manuscript. Michael Spector, L Brendan Young, and Nicole McGlynn engaged students in one of the respective seminars and reviewed drafts of the manuscript.

## Funding

The authors have nothing to report.

## Conflicts of Interest

The authors declare no conflicts of interest.
